# Working Together to Make the World a Healthier Place: Desiderata for the Pharmaceutical Industry

**DOI:** 10.1017/S096318011800049X

**Published:** 2019-01

**Authors:** KLAUS M. LEISINGER, KATE CHATFIELD

**Keywords:** research ethics, corporate responsibility, pharmaceutical industry, responsible research and innovation (RRI), low- and middle-income countries

## Abstract

Cross-sectorial, dynamic, and innovative partnerships are essential to resolve the challenges of humankind in the 21st century. At the same time, trust in each other’s integrity and good will is a precondition for the solution of any complex problem, and certainly for the success of the United Nations Sustainable Development Agenda. Experience shows that a nation’s economic and social success is at its greatest if, and when, there is cooperation and even cocreation involving a fair division of labor and responsibility among the different societal stakeholders. This paper uses Ralf Dahrendorf’s seminal work on obligations, as well as the European Commission’s Science with and for Society unit’s definition of responsible research and innovation (RRI), to motivate industry responsibilities to make the world a healthier place.

## 21st Century Challenges

The most recent (2017) *State of the Future* report from the Millennium Project^1^ paints a candid picture of the ongoing grand challenges that face humankind in the 21st century such as access to clean water, the rich-poor gap, sustainable development and climate change, population growth, increasing energy demands, and health issues. While recent efforts have yielded certain tangible improvements such as an increase in life expectancy and a decrease in child mortality, poverty, contagious disease, and illiteracy, serious challenges remain, in particular, increases in the global population and in environmental degradation.

As emphasized in the Millennium Project report, all of the challenges are transnational in nature and they demand transinstitutional solutions; they cannot be addressed by any single government or institution alone and will require collaborative action among governments, international organizations, corporations, universities, nongovernmental organizations, and individuals. There are clear indications, from this report and others, of increasing consensus about the global situation and the actions needed to address the colossal challenges facing humankind.

A highly significant development in this regard has been the agreement of the Sustainable Development Goals (SDGs), which call for a concerted global effort to transform our world by 2030.^2^ The Agenda 2030 for Sustainable Development is a nonbinding document released by the United Nations in 2015, after considerable deliberations and consultations amongst 193 member states and global civil society. The Agenda has set 17 SDGs with 169 targets. All 17 goals are directly related to the 2012 document *The Future We Want,*^3^ which was agreed at the Rio+20 Conference held in 2012. The United Nations Department of Economic and Social Affairs has noted:

The achievement of the 2030 Agenda for Sustainable Development and the Sustainable Development Goals will require all hands on deck. It will require different sectors and actors working together in an integrated manner by pooling financial resources, knowledge, and expertise. In our new development era with 17 intertwined Sustainable Development Goals and 169 associated targets as a blueprint for achieving the sustainable Future We Want, cross-sectorial and innovative multistakeholder partnerships will play a crucial role for getting us to where we need by the year 2030.^4^

Successful research and innovation, undertaken in a responsible manner, is a prerequisite for efforts to implement the SDGs. The necessity for cooperative efforts in research and innovation for successful implementation of the goals is clear: since the SDGs seek to address complex global problems that cannot be solved by single actors,^5^ multiple stakeholders must work together to find solutions and ensure that actions are taken. In particular, the “enhancement of North-South international cooperation on, and access to, science, technology and innovation” is a named target. It is part of SDG 17: “global partnerships for sustainable development”.^6^ In the European Union (EU), significant emphasis is placed upon “research and innovation” as a key solution to Europe’s economic and social problems and this has become an integral part of the Europe 2020 policy structure, in the form of the Innovation Union flagship.^7^

## Ethics Dumping

Progressive globalization in research and innovation may undoubtedly convey substantial benefits, but it can also provoke a multitude of sensitive ethical issues. For instance, in clinical trials, participant populations may differ greatly between regions in terms of usual standards of medical care, cultural beliefs and norms, levels of education and literacy, socioeconomic status, and access to legal systems. Ethical approval systems and research governance structures may vary widely between countries and between institutions.^8^ Consequently, there is a risk that research that is not permissible in northern, high-income countries will be conducted in southern, low- and middle-income countries (LMICs) where the legal and regulatory frameworks for research are not as stringent. The European Commission (EC) has highlighted this threat, pointing out that when research is conducted by European organizations outside the EU, there is a risk that, without proper compliance structures and follow-up, ethical standards may be neglected. The EC has termed this practice “ethics dumping.”^9^ (The term “dumping,” in the business context, is traditionally associated with predatory pricing policies. Large entities can afford to undercut local competitors for a given period in order to drive them out of the market.) In the context of research ethics, “ethics dumping” can mean *both* the purposeful exploitation of third country research participants and resources and exploitation based on insufficient ethics awareness. In exploitative relationships, peoples’ vulnerabilities are treated as opportunities to advance others’ interests or projects.^10^

The EU is currently funding actions to address the risk of ethics dumping from both public and private research, and one such action is the TRUST project.^11^ TRUST aims to foster adherence to high ethical standards in research globally and to counteract the practice of ethics dumping, or the application of double standards in research. The will to act in collaboration across national, institutional, political, religious, and ideological boundaries that is necessary to address today’s global challenges also requires cooperation on global ethics.^12^ However, any measure to improve global ethical research governance and adherence to high ethical standards in the long term will fail if highly important stakeholders are not involved in the process of its development. Without sharing “ownership” of the process and of the outcomes of projects such as TRUST, significant stakeholders’ motivation to engage in fair and ethical research practices and comply with “yet another” set of standards and procedures cannot be presumed.

The attainment of global health and wellbeing (SDG #3) sets highly aspirational targets including an end of the epidemics of AIDS, tuberculosis, malaria, and other communicable diseases by 2030. It also aims to achieve universal health coverage and to provide access to safe and effective medicines and vaccines for all. This will entail, inter alia, support for the research and development of vaccines and medicines for diseases that primarily affect LMICs. Since the private sector, particularly the research-based pharmaceutical industry, is the most important innovator of health care products, improving adherence to high ethical standards globally must involve this industry.

The research-based pharmaceutical industry has an enormous impact on global health due to its global reach and its impact on setting international standards. In particular, its influence upon ethical standards in clinical research in LMICs cannot be ignored; most large, multinational pharmaceutical corporations undertake a proportion of their research activities, including clinical trials, in LMICs. Hence, the “buy in” of pharmaceutical corporation efforts to counteract ethics dumping and improve ethical standards in multinational, cross-cultural, collaborative research efforts is imperative.

## The Trust Issue

It is undeniable that cross-sectorial, dynamic, and innovative partnerships are essential to resolve the challenges of humankind in the 21st century, but at the same time, trust in each other’s integrity and goodwill is a precondition for the solution of any complex problem and certainly for the success of the Sustainable Development Agenda. Without trust, as UN Secretary General António Guterres has said, we cannot face the difficult challenges for our world today.^13^ However, today’s overall global societal atmosphere is characterized by a lack of trust in fundamental institutions. Following their most recent (2017) *Trust Barometer*, global communications marketing firm Edelman concluded that “trust is in crisis around the world.”^14^ Edelman’s 17th annual survey included more than 33,000 individuals, across 28 countries, and found that *trust to do what is right* had declined broadly across business, government, nongovernmental organizations, and media; the largest-ever drop in trust since they began their tracking. The same poll revealed a lack of credibility in regard to both business *and* government leadership and, significantly, 82% of respondents were of the opinion that the pharmaceutical industry needs more regulation.

In the business sector, lack of trust arises when people either find out about indecent corporate behavior or perceive that harm of any kind has been inflicted, particularly on vulnerable human beings. Individual scandals can serve as a justification for more generalized criticisms, which further erode trust, and the internet abounds with examples of corporate crime. For the past 30 years, Washington, D.C., based Corporate Crime Reporter^15^ has been publishing cases of corporate crime in America; some recent reports involve members of the pharmaceutical industry. Following legal action, pharmaceutical companies have been ordered to pay millions of dollars to resolve allegations of violations of US acts like the Federal and State False Claims Act^16^ and the Federal Food, Drug and Cosmetic Act,^17^ as well as allegations of fraudulent behavior such as knowingly reporting to the government false and fraudulent prices of products.^18^ Without passing judgement on any particular cases, it is fair to say that the international pharmaceutical industry currently has a public image problem^19^ and such cases do not help to (re)build trust in the industry.

Given the heterogeneity of the pharmaceutical industry (research-based and generic companies, companies of different sizes, each with their own unique ethos and different types of leadership, etc.), generalizing judgments are inappropriate. The sheer size and complexity of multinational corporations makes the building of trust across all areas of the business challenging, even if a company is operating with integrity.^20^ From the perspective of many civil society activists, the fact that research endeavors in the private sector are usually driven by profit motives adds an additional layer of skepticism. In this context, for many stakeholders from civil society, academia, and the media, clinical trials in LMICs, as well as access to innovative medicines, are highly controversial subjects, overshadowed by a significant lack of trust towards the corporate actors involved.^21^ This poses a problem for all, not just the industry; the pharmaceutical industry is vital for collaborative efforts towards achievement of the SDGs and trust is fundamental for effective collaborations.

How can this problem be resolved? Between 56% and 65% of respondents in the previously mentioned Trust Barometer rated *ethical business practices* as one of the top five preconditions required to build trust in a company.^22^ Of course, “doing no harm” is the most important precondition for preventing the creation of distrust, but companies must also “do good” in order to build trust and they are increasingly “expected to do more,”^23^ particularly with regard to those who live in impoverished conditions all over the world.

Indeed, many pharmaceutical companies have taken it upon themselves to develop strategies for the cocreation of solutions for the SDGs, making public declarations about their contributions. Some have begun to report upon developments in their annual reports. For instance, for each of the 17 goals, *Roche* provides examples of efforts or agreed company goals; for goal 5, gender equality, the company aims to increase the percentage of women in leadership roles to 29% by 2020 (from 13% in 2009).^24^ *Pfizer* declares a commitment to help achieve all 17 SDGs, but in particular, seven of the goals are highlighted as being closely aligned to their mission as a research-based biopharmaceutical company.^25^ Working towards goal 6, clean water and sanitation, *Pfizer* describe public-private partnerships including the International Trachoma Initiative,^26^ as well as efforts to responsibly manage the company’s own water consumption and disposal. *Novartis* has developed several access to medicines programs and has taken steps towards achieving its Vision 2030 on Environmental Sustainability and 2020 targets. Overall, *Novartis* has managed to reduce its net greenhouse gas emissions by 18.7% since 2010. Development has also begun on an approach to capture, measure, and value the external economic, environmental, and social impacts created by the company’s activities.^27^ *Glaxosmithkline* expresses a long-term commitment to improving access to health care across the world. Since 2010, it has capped the prices of patented medicines and vaccines in the “least developed countries” at 25% of those in the EU5 (France, Germany, Italy, Spain, and the U.K.) as long as manufacturing costs are covered.^28^

From an institutional perspective, trust is a forward-looking metric. Unlike reputation, which is based on an organization’s historical behavior, trust is a predictor of credibility in the future.^29^ To rebuild trust and restore faith in the system, institutions must step outside of traditional business roles and work towards integrated operating models that put all stakeholders (humans, animals, and the environment), not just shareholders, at the center of their activities.^30^ The above examples demonstrate steps in this direction. In the context of clinical research, the stakeholders in each different location/environment may have their own unique perspectives, assumptions, needs, and expectations. In LMICs these can be heavily influenced by factors such as high disease burdens, low socioeconomic status, and poor environmental conditions, thereby increasing vulnerability to ethics dumping. These additional complexities and sensitivities demand additional care.

## A Practical Approach to Corporate Responsibility

**Figure 1.**
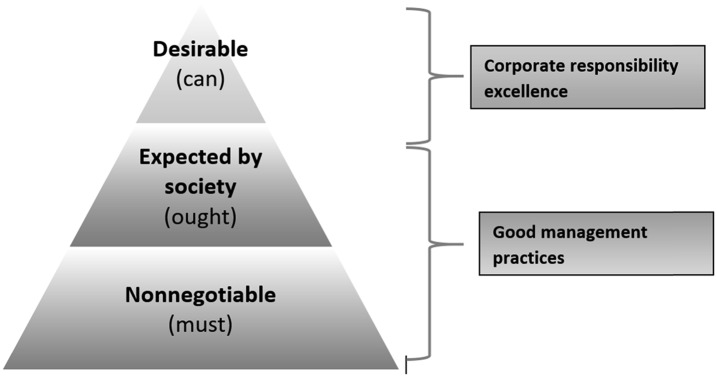
Hierarchy of Responsibility Levels.

The Ralf Dahrendorf model, as suggested in his seminal work *Homo Sociologicus,*^31^ is helpful for the gradation of corporate responsibility areas and can be visualized as a pyramid of responsibility levels (**Figure 1**). Each level consists of specific moral duties with their specific grade of liability as follows:

• **Level 1. Must-expectation** (*Muss-Erwartung*): no positive reward for compliance but negative sanction for noncompliance
• **Level 2. Ought-expectation** (*Soll-Erwartung*): sympathy/goodwill for compliance but societal rejection/exclusion for noncompliance
• **Level 3. Can-expectation** (*Kann-Erwartung*): appreciation/esteem for compliance and antipathy for noncompliance

The ***must*** dimension forms the base of the pyramid and comprises nonnegotiable corporate duties which include, for example, compliance with national law and regulation and avoidance of deception or fraud. This includes protection of the environment, as well as the health and safety of employees, customers, and neighbors according to applicable law. Other duties that lie within this domain are obligations to meet the expectations of shareholders and employees; shareholders expect a fair return on their investment and employees expect fair wages. Companies also add value to society by providing products and services that meet customer needs or enhance their quality of life. In the case of pharmaceutical corporations, this includes medicines and vaccines that enhance health and wellbeing.

To compete with integrity, businesses *must* achieve business success without inflicting collateral social, ecological, or political damage. This demands awareness of risk factors for such damage. Ethical challenges and dilemmas do not arise in a vacuum, they always happen in a specific social, economic, cultural, political *context*. To manage risks effectively, one has to know them in the specificity of their context. For this reason, pharmaceutical companies *must* conduct ex ante due diligence processes before a clinical trial is conducted in LMICs. Only ex ante due diligence assessments enable responsible actors to identify and account for potential adverse/undesirable impacts on populations and to take steps to prevent, mitigate, and address them.

Due diligence includes assessing actual and potential vulnerabilities and impacts, integrating and acting upon the findings, tracking responses, and communicating how impacts are addressed. The initial steps in conducting an ex ante due diligence may be

• to identify and assess the *nature* of the actual and potential adverse impacts of business or research activities (for example, all the processes and procedures involved in a clinical trial and its conduct in this environment);
• to identify *who/what* may be affected and *how* (for example, the participants, local researchers, local health personnel, and local communities).

It is then possible to decide on accompanying measures to avoid or mitigate potentially undesirable impacts on those identified. These processes should involve meaningful consultation with potentially affected groups as well as other relevant stakeholders. Ex ante due diligence is absolutely nonnegotiable as ignorance of the local context should never be used as an excuse for the exploitation of vulnerable populations.

The ***ought to*** dimension comprises acts of responsibility that go beyond legal compliance in a national context. In some countries, where the quality of law is state-of-the-art and enforced effectively, adherence to national laws and regulations goes a long way towards satisfying the requirements of responsible corporate conduct. However, in regions where this is not the case, responsible companies will exceed legal minima by applying higher corporate norms, for example, through the use of state-of-the-art environmental technology and social policies, *even where* local law would permit lower standards.

In the case of pharmaceutical corporations, the *ought to* responsibilities can include a wide range of activities such as support for broader global goals (like the SDGs) or support for within-country health and development goals that are relevant to the local context. Another *ought to* responsibility in the context of clinical trials in LMICs includes taking additional steps to maintain accountability and avoid any watering down of accountability through outsourcing. In collaborative work, important and sensitive tasks are often outsourced to third parties, who may then further outsource parts of the work to other actors. For example, in India, it is estimated that more than three hundred contract research organizations have been established, within a short time frame, to serve the requirements of pharmaceutical companies. Personnel within these organizations, many of whom are not properly qualified, may act as the local principal investigator in clinical studies, taking on responsibility for local management. This has resulted in serious ethical infringements such as failure to seek informed consent, recruitment of unsuitable participants, failure to manage side effects from the investigative treatment, and the receipt of substantial incentives for timely recruitment.^32,33^

The totality of the *must* and the *ought* domains constitute “good management practices.” Acting upon these responsibilities will help to ensure a thriving business. Hence, there is a clear element of enlightened self-interest for corporate responsibility at these levels.

On the other hand, the ***can*** dimension of corporate responsibility at the top of the pyramid is not obligatory either by law or by industry standards. It encompasses socially desirable practices and investments such as pro bono research for tropical and poverty-related diseases. Corporate philanthropy belongs to this level and is usually not bound to any direct company advantages or measurable financial return. In the context of pharmaceutical company work in LMICs, an example of a *can* activity might be the opening of access to unused product patents for the development of medicines for use in low-income settings. Currently, a substantial proportion of patents lay unused,^34^ yielding no return on what may have been years of work and significant economic investment. Redevelopment of these abandoned compounds could build on this prior work, meaning a potentially shorter path to market and focusing on addressing unmet medical needs.^35^ Pharmaceutical companies can chose to open access to such patents for not-for-profit institutions and provide a free license when suitable medicines are developed.

Management has no option when it comes to adhering to laws and regulations, and “good management practices” may be driven by enlightened self-interest, but corporate citizenship deliverables above and beyond a certain standard remain at the sole discretion of management. From a purely economic point of view, it could be argued that every dollar spent on corporate responsibility beyond legal requirements and basic standards of decency is a dollar diverted from potentially profit-generating activity. This is why, ultimately, the top management of every company has to draw the line on what it can assume responsibility for. While dialogue with open-minded stakeholders will help to sharpen awareness about social, political, and environmental problems, the ultimate decision on how far a company extends its “responsibility frontier” remains the prerogative of informed top management.

## Responsible Research and Innovation

The subject of corporate responsibility occupies a prominent position on the global corporate agenda and has significant importance as an area of business practice and academic inquiry.^36,37^ However, in comparison to other business activities, company research and innovation activities have received scant attention in the corporate responsibility discourse. This is changing rapidly; current EU policy specifically underlines the importance of research and innovation in addressing grand challenges and, more generally, highlighting the need for a *responsible* approach to research and innovation activities.^38^ In this context, the EU is promoting the concept of responsible research and innovation (RRI) as a key governance framework that underpins its considerable investment in research and technology development.

As a field of study and practice, RRI has gained prominence over the last decade, its aim being to ensure that research and innovation activities are socially acceptable, desirable, and sustainable.^39^ In general terms, RRI can be thought of as a move towards greater inclusion and responsiveness in the governance of research and innovation.^40^ While discussion and debate about the precise nature of RRI is ongoing,^41^ there are certain recurring themes that emerge from the RRI discourse. These include matters such as the need for alignment of research and innovation with societal needs, the need to anticipate and be responsive to ethical, environmental and societal concerns, and the need to enhance these efforts through engagement with a broad range of stakeholders.^42,43^

As the largest funder of research in Europe, the EC has a significant influence on research policy and it has adopted RRI as both a subject of study and a condition of funding.^44^ The concept of RRI is integral to the EC’s Horizon 2020 research framework program (2014-2020), and during its lifetime, Science with and for Society is funding research activities worth €460 million. In the EC’s view, RRI consists of a number of pillars that correspond to established research policy areas: civil society engagement, gender equality, science education, open access, ethics compliance, and governance.^45,46^ The question of how such goals can be achieved is typically answered with a reference to grand challenges.^47^ Where research addresses such grand challenges, it is deemed to be in the public interest and to fulfill the acceptability and desirability criteria of research.

The EC support for RRI may be laudable, but the promotion of RRI has, until recently, focused predominantly on publicly funded research, omitting a substantial proportion of company-based innovation activities.^48^ Given that the vast majority of research and innovation activities occur in the private sector, such policies need to be sensitive to specific industry-related challenges, such as tensions between the need for short-term profit and long-term stability and corporate research cultures. Additionally, RRI is, first and foremost, a European initiative, and while there have been attempts to promote RRI worldwide, including in the United States, China, Japan, India, Australia, and South Africa,^49^ the potential for global uptake and impact remains uncertain. In spite of its relative infancy and ongoing development, the business sector, including pharmaceutical companies, might benefit greatly from engagement with RRI.

Through engagement with RRI, pharmaceutical companies may find ways to move on from purely *must* and *ought to* levels of corporate responsibility to more effective and efficient adoption of *can* practices. The EC makes this clear with their declaration that RRI implies better alignment of the processes and outcomes of research and innovation with the values, needs and expectations of society,^50^ and, by inference, greater alignment with the SDGs. RRI extends a more conventional ethical review of research, and can be viewed as “creating opportunity.” The adoption of RRI compels us to reflect upon the sort of future(s) we want science and technology to bring to the world and how the aims and objectives of research and innovation can be identified in an ethical, inclusive, and equitable manner.^51^

Recent efforts to engage with the private sector^52^ to explore the perceived relevance for them of RRI have resulted in highly positive feedback and the development of tools that are specifically designed to aid practical implementation and action. For example, Stahl et al.^53^ have developed an easily applied RRI maturity model that is linked to corporate research and development processes. Such a model could be useful for organizations to understand and reflect on their current practice, to compare their activities with good industry practice, and to see where they could take action to achieve corporate responsibility excellence in their research and innovation activities.

## Moving Forwards: Cocreation not Demarcation

When analyzing the responsibility performance of individual actors working in LMICs, we find a number of nonnegotiable good practices or duties that each actor is responsible for. Not everything that is legally allowed can be considered legitimate, and in the spirit of internationally accepted norms, the corporate responsibility of multinational pharmaceutical giants often goes beyond the letter of the law.^54^ In a low-income environment, this can mean taking charge of, and investing time and resources in, the management of issues that would not be considered as corporate duties in other environments (like the opening of access to unused product patents ).

No one stakeholder can assume responsibility for all necessary steps towards sustainable development. While those with “broader shoulders” should carry a higher load, a division of labor, as envisaged by the Agenda 2030, is inevitable. A nation’s economic and social success is often at its greatest when there is cooperation involving a fair division of labor and responsibility among the different societal stakeholders on the basis of shared values.^55^ It is constructive cocreation, not ideological demarcation, that leads to socio-economic progress on the basis of shared values.

Social systems of poverty consist of a number of undesirable conditions for work and life (low outputs and incomes, low productivity and conditions of production, low quality of life, low aspirations toward life and work, unfavorable institutions, and inappropriate policies); these have been well described by Nobel Laureate Gunnar Myrdal.^56^ Such conditions are caught in a system of circular interdependence; a change in one will cause changes in the others, which leads to self-reinforcing processes of both a positive and a negative kind.

When discussing the ethical framework for clinical trials or other research in this context, it is reasonable to recall that a history of colonialism has much to do with the current state of poverty as well as the governance structures of many LMICs in which clinical trials are conducted. While it would be misguided to attribute all socioeconomic evils and political irresponsibility in poor countries to past colonialism, it remains a fact that colonialism and its consequences have affected the nature of political and economic power, the structure of government and, in some cases, cultures of corruption. It also boosts the sensitivities of people who have been exploited by foreigners in the past. The “lows” referred to by Myrdal have their origin not in the local people’s unwillingness to work hard but in postcolonial institutions and structures that continue to siphon resources away from the local areas, whether to wealthy local elites or to people in other countries.

Working in social systems of poverty requires special care and diligence from corporations. As articulated in this paper, this demands attention to corporate responsibility at levels above the purely functional *must* requirements and even the desirable *ought to* responsibilities. Pharmaceutical corporations have a pivotal role to play in the cooperative action that is needed to achieve the ambitious SDGs, and there is clear evidence that some enlightened corporate leaders are already engaging with these responsibilities. However, this is not yet ubiquitous across all corporations, and the industry is still plagued by a general lack of trust. Trust is vital for effective collaborative actions, but it is also a valuable asset for all institutions, and ongoing trust-building activities should be one of the most important strategic priorities for every organization.^57^ The adoption of RRI could prove to be an effective way of building trust in the research and innovation activities of pharmaceutical companies with the assurance that such activities are socially acceptable, desirable, and sustainable. Ultimately, to reach the goal of global health and wellbeing, pharmaceutical corporations must strive for corporate responsibility excellence, as nothing less will suffice.

## Notes

1. GlennJC, FlorescuE, and the Millenium Project team. The State of the Future, version 19; 2017; available at http://107.22.164.43/millennium/SOF2017-ExecSumm-front_matter.pdf (last accessed 3 Oct 2017).

2. United Nations. Sustainable Development Goals; available at http://www.un.org/sustainabledevelopment/ (last accessed 1 Oct 2017).

3. European Commission. The Future We Want; available at https://www.eea.europa.eu/policy-documents/the-future-we-want-2013declaration (last accessed 3 Oct 2017).

4. United Nations. Multi-stakeholder Partnerships and Voluntary Commitments; available at https://sustainabledevelopment.un.org/sdinaction (last accessed 4 Oct 2017).

5. VoegtlinC, SchererAG. Responsible innovation and the innovation of responsibility: Governing sustainable development in a globalized world. Journal of Business Ethics 2017;143(2):227–43.

6. United Nations. Goal 17; available at http://www.un.org/sustainabledevelopment/globalpartnerships/ (last accessed 1 Oct 2017).

7. European Commissions. Innovation Union; available at http://ec.europa.eu/research/innovation-union/index_en.cfm?pg=intro (last accessed 1 Oct 2017).

8. ChatfieldK, SchroederD, LeisingerK, van NiekerkJ, MunuoN, WynbergR, et al. Generic Risks of Exporting Non-ethical Practices, a Report for TRUST; 2016; available at http://trust-project.eu/deliverables-and-tools/RisksMatrix. (last accessed 9 Oct 2017).

9. European Commission. Horizon 2020, the EU Framework Programme for Research and Innovation: Ethics; 2016; available at https://ec.europa.eu/programmes/horizon2020/en/h2020-section/ethics (last accessed 6 Oct 2017).

10. SchroederD, CookJ, HirschF, FenetS, MuthuswamyV. Ethics Dumping—Case Studies from North-South Research Collaborations. New York: SpringerBriefs in Research and Innovation Governance; 2017.

11. Trust-project.eu. TRUST – Equitable Research Partnerships; c 2015-2017 [cited 2017 Jul 26]; available at http://trust-project.eu/ (last accessed 17 Sept 2018).

12. See Note 1, Glenn et al. 2017

13. Lee J. 14 quotes from UN leaders on humanity’s biggest challenges. *United Nations Foundation Blog*; 2017; available at http://unfoundationblog.org/14-quotes-from-un-leaders-on-humanitys-biggest-challenges/ (last accessed 1 Oct 2017).

14. Edelman.com. *The 2017 Edelman Trust Barometer*; c2017 [cited 2017 Oct 3]; available at http://www.edelman.com/global-results/ (last accessed 4 Oct 2017).

15. *Corporate Crime Reporter*; c2017 [cited 2017 Oct 1]; available at https://www.corporatecrimereporter.com/ (last accessed 4 Oct 2017).

16. Corporate Crime Reporter. Shire Pharmaceuticals to Pay $350 Million Dollars to Settle False Claims; available at https://www.corporatecrimereporter.com/news/200/shire-pharmaceuticals-to-pay-350-million-to-settle-false-claims-charge/ (last accessed 4 Oct 2017).

17. Corporate Crime Reporter. Sanofi Unit Genzyme Gets Prosecution Deferred, to Pay $32.5 Million to Resolve Criminal Charge; available at https://www.corporatecrimereporter.com/news/200/sanofli-unit-genzyme-gets-prosecution-deferred-to-pay-32-5-million-to-resolve-criminal-charge/ (last accessed 4 Oct 2017).

18. Corporate Crime Reporter. Wyeth and Pfizer to Pay $784.6 Million to Resolve False Claims Charge; available at https://www.corporatecrimereporter.com/news/200/wyeth-and-pfizer-to-pay-784-6-million-to-resolve-false-claims-charge/ (last accessed 4 Oct 2017).

19. BraithwaiteJ. Corporate Crime in the Pharmaceutical Industry. London: Routledge Revivals; 2013.

20. LeisingerKM. Corporate leadership in times of public distrust In: Coutinho de ArrudaMC, RokB, eds. Understanding Ethics and Responsibilities in a Globalizing World, The International Society of Business, Economics and Ethics, Book Series 5. New York: Springer; 2016:15–40.

21. Theguardian.com. Ethics Left Behind as Drug Trials Soar in Developing Countries; July 4 2011 [cited 2017 Oct 2]; available at https://www.theguardian.com/global-development/2011/jul/04/ethics-left-behing-drug-trials-developing (last accessed 4 Oct 2017).

22. See note 14, Edelman 2017.

23. See note 14, Edelman 2017.

24. Roche.com. *Sustainable Development Goals*; c2017 [cited 2017 Oct 1]; available at http://www.roche.com/sustainability/our_reporting/un-sdgs.htm (last accessed 4 Oct 2017).

25. Pfizer.com. *Annual Report 2016*; c2017 [cited 2017 Oct 1]; available at https://www.pfizer.com/files/investors/financial_reports/annual_reports/2016/our-business/sustainable-development-goals-sdgs/index.html (last accessed 4 Oct 2017).

26. Trachoma.org. *International Trachoma Initiative: A program of the Task Force for Global Health*; c2017 [cited 2017 Oct 10]; available at http://www.trachoma.org/ (last accessed 4 Oct 2017).

27. Novartis.com. *Progress Towards Access and Environmental Goals 2016*; c2017 [cited 2017 Oct 1]; available at https://www.novartis.com/news/media-releases/novartis-highlights-progress-towards-access-and-environmental-goals-2016 (last accessed 4 Oct 2017).

28. GlaxoSmithKline. *Access to Healthcare*; c2017 [cited 2017 Oct 1]; available at https://www.gsk.com/en-gb/responsibility/health-for-all/access-to-healthcare/ (last accessed 4 Oct 2017).

29. See note 14, Edelman 2017.

30. FreemanRE, VelamuriSR. A new approach to CSR: Company stakeholder responsibility In: KakabadseA, MorsingM, eds. Corporate Social Responsibility. London: Palgrave Macmillan; 2006:9–23.

31. DahrendorfR. Homo Sociologicus. Abingdon on Thames: Routledge and Kegan Paul books; 1973.

32. BhanA. Clinical trial ethics in India: One step forward, two steps back. Journal of Pharmacology & Pharmacotherapeutics 2012;3(2):95–7.2262908010.4103/0976-500X.95501PMC3356974

33. YeeA. Regulation failing to keep up with India’s trials boom. The Lancet 2012;379(9814):397–98.10.1016/s0140-6736(12)60172-x22312640

34. TorrisiS, GambardellaA, GiuriP, HarhoffD, HoislK, MarianiM. Used, blocking and sleeping patents: Empirical evidence from a large-scale inventor survey. Research Policy 2016;45(7):1374–85.

35. ChesbroughH, ChenEL. Using inside-out open innovation to recover abandoned pharmaceutical compounds. Journal of Innovation Management. 2015:3(2):21–32.

36. DuS, BhattacharyaC, SenS. Maximizing business returns to corporate social responsibility (CSR): The role of CSR communication. International Journal of Management Reviews 2010;12(1):8–19.

37. SmithC, LenssenG. Mainstreaming Corporate Responsibility. Hoboken, NJ: John Wiley and Sons; 2009.

38. European Commission. Options for Strengthening Responsible Research and Innovation; 2013; http://ec.europa.eu/research/science-society/document_library/pdf_06/options-for-strengthening_en.pdf (last accessed 4 Oct 2017).

39. StilgoeJ, OwenR, MacnaghtenP. Developing a framework for responsible innovation. Research Policy 2013;42:1568–80.

40. See note 37, Stilgoe, Owen, Macnaghten*, 2013*.

41. ChatfieldK, IatridisK, StahlBC, PaspallisN. Innovating responsibly in ICT for ageing: Drivers, obstacles and implementation. Sustainability 2017;9:971.

42. OwenR, StilgoeJ, MacnaghtenP, GormanM, FisherE, GustonDA. Framework for responsible innovation In: Responsible Innovation: Managing the Responsible Emergence of Science and Innovation In Society*.* Hoboken, NJ: John Wiley and Sons Ltd; 2013:27–50.

43. Von SchombergR. A vision of responsible research and innovation In: Responsible Innovation: Managing the Responsible Emergence of Science and Innovation in Society*.* Hoboken, NJ: John Wiley and Sons Ltd; 2013:51–74.

44. European Commission. Responsible Research and Innovation—Europe’s Ability to Respond to Societal Challenges; 2012; available at http://ec.europa.eu/research/science-society/document_library/pdf_06/responsible-research-and-innovation-leaflet_en.pdf (last accessed 22 Sept 2017).

45. See note 39, Chatfield, Iatridis, Stahl, Paspallis 2017.

46. See note 42, European Commission 2012.

47. BodenM, JohnstonR, ScapoloF. The role of FTA in responding to grand challenges: A new approach for STI policy? Science and Public Policy 2012;39(2):5.

48. IatridisK, SchroederD. Responsible Research and Innovation in Industry: The Case for Corporate Responsibility Tools. Cham, Switzerland: Springer; 2016:104.

49. DavisM, LaasK. “Broader impacts” or “Responsible research and innovation”? A comparison of two criteria for funding research in science and engineering. Science and Engineering Ethics 2014;20(4):963–83.2415515910.1007/s11948-013-9480-1

50. European Commission. Horizon 2020, the EU Framework Programme for Research and Innovation: Ethics; available at https://ec.europa.eu/programmes/horizon2020/en/h2020-section/ethics (last accessed 30 Aug 2017).

51. See note 40, Owen et al. 2013.

52. Responsible-industry.eu. *Responsible Industry project*; c2014 [cited 2017 Oct 4]; available at http://www.responsible-industry.eu/ (last accessed 4 Oct 2017).

53. StahlBC, ObachM, YaghmaeiE, IkonenV, ChatfieldK, BremA. The responsible research and innovation (RRI) maturity model: Linking theory and practice. Sustainability 2017;9(6):1036. doi: 10.3390/su9061036

54. LeisingerKM. Corporate responsibilities in a world of cultural diversity and pluralism of values. Journal of International Business Ethics 2015;8(2):9.

55. LeisingerKM. Corporate leadership in times of public distrust In: de ArrudaMCC, RokB, eds. Understanding Ethics and Responsibilities in a Globalizing World. New York: Springer International Publishing; 2016:15–40.

56. MyrdalG. Inquiry into the Poverty of Nations. Harmondsworth: Penguin 1968:1840.

57. See note 14, Edelman 2017.

